# Obtusifolin, an Anthraquinone Extracted from *Senna obtusifolia* (L.) H.S.Irwin & Barneby, Reduces Inflammation in a Mouse Osteoarthritis Model

**DOI:** 10.3390/ph14030249

**Published:** 2021-03-10

**Authors:** Jiho Nam, Dong-Won Seol, Choong-Gu Lee, Gabbine Wee, Siyoung Yang, Cheol-Ho Pan

**Affiliations:** 1 Department of Biomedical Sciences, Ajou University Graduate School of Medicine, Suwon 16499, Korea; yjtnng3043@ajou.ac.kr; 2 Department of Pharmacology, Ajou University School of Medicine, Suwon 16499, Korea; 3 Degenerative InterDiseases Research Center, Ajou University School of Medicine, Suwon 16499, Korea; 4 Laboratory Animal Center, Daegu-Gyeongbuk Medical Innovation Foundation (DGMIF), Daegu 41061, Korea; seol@dgmif.re.kr; 5 Natural Product Informatics Research Center, Korea Institute of Science and Technology, Gangneung 25451, Korea; cglee0708@kist.re.kr; 6 Division of BioMedical Science & Technology, KIST School, Korea University of Science and Technology (UST), Gangneung 25451, Korea

**Keywords:** obtusifolin, osteoarthritis, inflammation, NF-κB

## Abstract

Osteoarthritis (OA) is an age-related degenerative disease that causes cartilage dysfunction and inflammation. Obtusifolin, an anthraquinone extracted from *Senna obtusifolia* (L.) H.S.Irwin & Barneby seeds, has anti-inflammatory functions; it could be used as a drug component to relieve OA symptoms. In this study, we investigated the effects of obtusifolin on OA inflammation. In vitro, interleukin (IL)-1β (1 ng/mL)-treated mouse chondrocytes were co-treated with obtusifolin at different concentrations. The expression of matrix metalloproteinase (Mmp) 3, Mmp13, cyclooxygenase 2 (Cox2), and signaling proteins was measured by polymerase chain reaction and Western blotting; collagenase activity and the PGE_2_ level were also determined. In vivo, OA-induced C57BL/6 mice were administered obtusifolin, and their cartilage was stained with Safranin O to observe damage. Obtusifolin inhibited Mmp3, Mmp13, and Cox2 expression to levels similar to or more than those after treatment with celecoxib. Additionally, obtusifolin decreased collagenase activity and the PGE_2_ level. Furthermore, obtusifolin regulated OA via the NF-κB signaling pathway. In surgically induced OA mouse models, the cartilage destruction decreased when obtusifolin was administered orally. Taken together, our results show that obtusifolin effectively reduces cartilage damage via the regulation of MMPs and Cox2 expression. Hence, we suggest that obtusifolin could be a component of another OA symptom reliever.

## 1. Introduction

Osteoarthritis (OA) is a degenerative disease caused by cartilage destruction or joint cartilage inflammation and usually occurs in elderly individuals [1,2]. The molecules involved in cartilage destruction and inflammation include matrix metalloproteinases (MMPs) and cyclooxygenase 2 (Cox2), which are catabolic factors [3,4]. The expression of these factors is increased by proinflammatory cytokines (interleukin (IL)-1β, tumor necrosis factor-α, and IL-17) [5,6]. Among the MMPs, Mmp3 and Mmp13 degrade the extracellular matrix by inducing aggrecanase and collagenase activities and increasing the PGE_2_ level, causing cartilage degradation and, thereby, resulting in OA [5,7]. An increased Cox2 level is involved in the expression of MMPs and leads to inflammation [5,8].

Several signaling pathways involved in OA are regulated by these proinflammatory cytokines. Among them, the NF-κB signaling pathway, which regulates the expression of Mmp3, Mmp13, and Cox2, is targeted in OA treatment [9,10,11]. Blocking the activation of this pathway suppresses the expression of catabolic factors and prevents OA progression [12,13,14].

Most drugs prescribed to patients control inflammation and prevent disease progression [1,15]. Among the potential ingredients of anti-OA medicine, anthraquinones have anti-inflammatory and anti-arthritic properties [16,17,18,19]. For example, anthraquinones downregulate the expression of inflammation-related molecules, such as Cox2 [19,20,21]. Thus, several studies have investigated whether anthraquinones can prevent OA progression [19,22,23].

Obtusifolin, an anthraquinone extracted from the seeds of *Senna obtusifolia* (L.) H.S.Irwin & Barneby, inhibits high-glucose-induced mitochondrial apoptosis, attenuates memory impairment, and suppresses breast cancer metastasis to bone [24,25,26]. Additionally, obtusifolin inhibits the NF-κB pathway in airway epithelial cells [27]. However, to date, there have been no reports on the association between obtusifolin and OA.

Considering that obtusifolin can control inflammation and regulate the NF-κB pathway in some cells, we hypothesized that obtusifolin would have a therapeutic effect on OA. Therefore, in this study, we investigated whether obtusifolin prevented OA progression via the regulation of Mmp3, Mmp13, and Cox2 through the NF-κB pathway. The results of this study can indicate the potential of obtusifolin as a component of anti-OA drugs.

## 2. Results

### 2.1. Obtusifolin Does Not Affect the Viability of Chondrocytes

**Figure 1:**
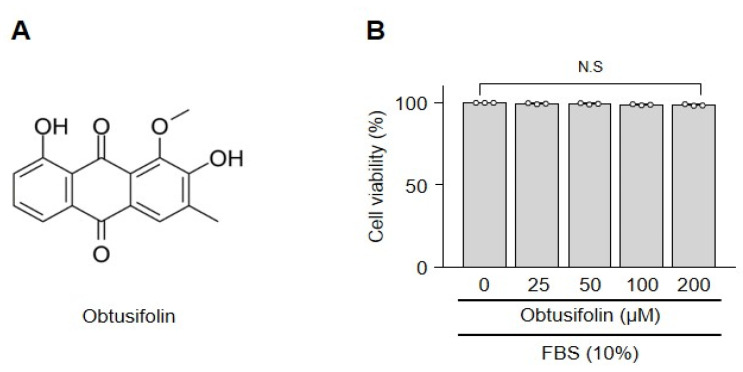
Viability of chondrocytes after obtusifolin treatment. The structure of obtusifolin (**A**). Chondrocytes were treated with obtusifolin for 24 h in a dose-dependent manner, and viability was measured using the lactate dehydrogenase assay (**B**). Data were analyzed using the one-way ANOVA, and values of each group (*n* = 3) are shown as mean ± SEM; N.S, nonsignificant.

We measured cytotoxicity using the lactate dehydrogenase assay to determine whether obtusifolin affected cartilage chondrocytes. Obtusifolin (Figure 1A) treatment at the indicated concentrations for 24 h did not reduce the viability of chondrocytes (Figure 1B).

### 2.2. Obtusifolin Inhibits Factors Involved in OA Pathogenesis

**Figure 2:**
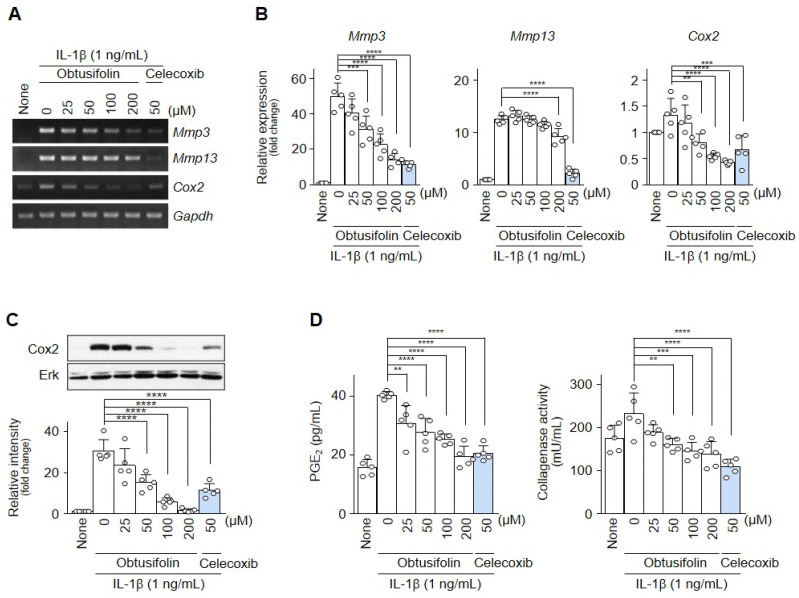
Obtusifolin downregulated Mmp3, Mmp13, and Cox2 expression in chondrocytes. Chondrocytes treated with IL-1β (1 ng/mL) for 24 h were co-treated with obtusifolin at the indicated concentrations and celecoxib (50 μM/mL) (**A**–**C**). The expression levels of Mmp3, Mmp13, and Cox2 were determined by RT-PCR (**A**) and qRT-PCR (**B**). Protein levels were determined by Western blotting (**C**, upper) and densitometry (**C**, lower). Collagenase activity and PGE_2_ level were measured at each concentration (**D**). Data were analyzed using the one-way ANOVA. The values of each group (*n* = 5) are shown as mean ± SEM; ** *p* < 0.01, *** *p* < 0.001, and **** *p* < 0.0001.

IL-1β (1 ng/mL)-treated chondrocytes were treated with different concentrations of obtusifolin and celecoxib (50 μM/mL). At the transcript and protein levels, obtusifolin dose-dependently reduced Mmp3, Mmp13, and Cox2 expression to levels similar to or more than those resulting from the effect of celecoxib (Figure 2A–C). In addition, obtusifolin dose-dependently reduced collagenase activity and the PGE_2_ level (Figure 2D). We also confirmed that the effect of obtusifolin on collagenase activity and the PGE_2_ level was similar to that after the addition of celecoxib.

### 2.3. Obtusifolin Regulates OA through the NF-κB Signaling Pathway 

**Figure 3:**
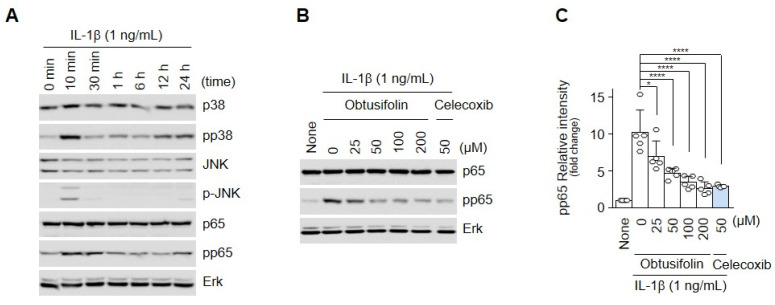
Obtusifolin regulates IL-1β-induced NF-κB phosphorylation. Chondrocytes were treated with IL-1β (1 ng/mL) for the indicated time. After harvesting, signaling proteins were quantified by Western blotting (**A**). Chondrocytes were treated with obtusifolin at the indicated concentrations for 24 h, and IL-1β (1 ng/mL) was added 10 min before harvest. pp65 was detected by Western blotting (**B**) and densitometry (**C**). Erk was used as the loading control in Western blotting. Data of each group (*n* = 5) are shown as mean ± SEM; * *p* < 0.05 and **** *p* < 0.0001.

OA is induced by inflammation or cartilage degradation through specific signaling pathways [5,28]. When chondrocytes are exposed to proinflammatory cytokines such as IL-1β, cell-signaling molecules including p38, JNK, and p65 are activated [29,30,31,32]. Western blotting (WB) showed that p38, JNK, and p65 were phosphorylated after the IL-1β treatment (1 ng/mL; Figure 3A). However, as the concentration of obtusifolin increased, the levels of phosphorylated P65 decreased, as determined by WB and densitometry (Figure 3B,C).

### 2.4. Effects of Obtusifolin In Vivo and Summary of Mechanism 

**Figure 4:**
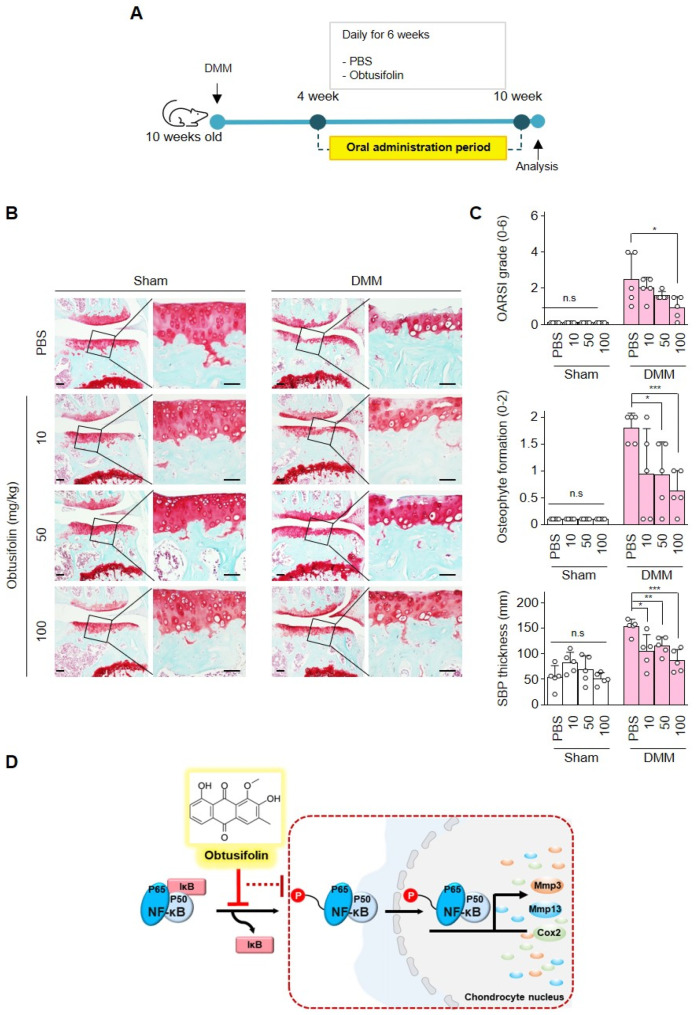
Oral administration of obtusifolin inhibits cartilage degradation. DMM(Destabilization of the medial meniscus)-induced osteoarthritic (OA) mice were administered PBS or obtusifolin (10, 50, and 100 mg/kg) daily for 6 weeks after 4 weeks of surgery (**A**). Safranin O staining shows the degree of cartilage destruction (**B**). Scale bar: 50 μm (left) and 100 μm (right). Cartilage destruction was scored by Osteoarthritis Research Society International (OARSI), subchondral bone thickness, and osteophyte formation (**C**). A graphical summary of the inhibition mechanism of obtusifolin (**D**). Data were analyzed using the one-way ANOVA and Student’s *t*-test. Values of each group (*n* = 5) are shown as mean ± SEM; * *p* < 0.05, ** *p* < 0.01, and *** *p* < 0.001.

The effect of obtusifolin was determined by DMM (Destabilization of the Medial meniscus) surgery and sham-operated surgery in mice. Obtusifolin and PBS, as the control, were orally administered to mice every day for 6 weeks after 4 weeks of DMM surgery. After 6 weeks of oral administration, the degree of cartilage damage was analyzed (Figure 4A). The cartilage of the sham-operated mice was intact, and the degree of cartilage destruction was dose-dependently reduced in the DMM mouse model (Figure 4B). The Osteoarthritis Research Society International (OARSI) grade, osteophyte formation, and subchondral bone plate thickness were also dose-dependently decreased by obtusifolin (Figure 4C). These results suggest that obtusifolin can reduce cartilage damage even in vivo. Based on our results, the mechanism of obtusifolin in OA is graphically presented (Figure 4D). Obtusifolin prevented cartilage damage via the inhibition of the phosphorylation of proteins involved in the NF-κB signaling pathway.

## 3. Discussion

MMPs and Cox2 induce cartilage destruction and inflammation in OA [3,5]. These catabolic factors are primarily activated downstream of proinflammatory cytokines [5,6]. Currently, nonsteroidal anti-inflammatory drugs (NSAIDs) are prescribed for patients with OA; however, these can cause various adverse effects owing to the toxicity of this class of drugs [33,34,35]. A representative NSAID for patients with OA is celecoxib, a selective Cox2 inhibitor, which is prescribed to control inflammation [36,37,38].

Obtusifolin, a compound belonging to the anthraquinone family, is present in *S. obtusifolia* [39]. In the USA, Africa, and Asia, *S. obtusifolia* is considered a remedy for several diseases such as rheumatism [40]. Reportedly, several anthraquinone substances are present in the seeds of *S. obtusifolia*, of which aurantio-obtusin is the most abundant [39]. Furthermore, other substances such as rhein, aloe-emodin, and emodin are also known to have arthritis-related anti-inflammatory properties [19,41,42,43]. These anthraquinones and their derivatives are commonly used as pharmaceutical ingredients [16,19]. Obtusifolin is the second most abundant compound in *S. obtusifolia*; it has been reported to suppress cancer metastasis, attenuate memory impairment, and reduce mitochondrial apoptosis [24,25,26,39]. However, the function of obtusifolin in OA is currently unknown. As studies have shown that anthraquinones have anti-inflammatory effects [16,19,41,42,43] and the NF-κB pathway is regulated by obtusifolin [27], we hypothesized that obtusifolin would have a sufficient OA inhibitory effect.

We first confirmed that obtusifolin did not affect cell survival, and then investigated the expression of OA catabolic factors. Proinflammatory cytokines such as IL-1β degrade the extracellular matrix of chondrocytes via the activation of Mmp3, Mmp13, and Cox2 [5]. In vitro, the increased expression of Mmp3, Mmp13, and Cox2 after IL-1β treatment decreased after the addition of the indicated concentrations of obtusifolin. Moreover, the expression of Mmp3, Mmp13, and Cox2 decreased to a level similar to or more than that after the addition of celecoxib.

Several signaling pathways induce OA via proinflammatory cytokines, which are involved in inflammation and cartilage degradation [28,30,31]. Cell signaling is activated by the phosphorylation of proteins related to signaling pathways by cytokines. In particular, obtusifolin has been reported to inhibit NF-κB signaling in airway epithelial cells [27], and this signaling pathway induces OA in chondrocytes [9,10,11]. The in vitro results confirmed that obtusifolin regulates this cell-signaling pathway under OA mimic conditions. The phosphorylation of NF-κB induced by IL-1β was decreased by obtusifolin treatment. Therefore, obtusifolin was confirmed to regulate OA through this pathway.

These results indicate that obtusifolin inhibits the activity of catabolic factors by inhibiting NF-κB signaling; thus, we expected that obtusifolin would prevent cartilage destruction in vivo.

OA can be induced in vivo using DMM, which enables the degree of OA progression to be measured [39]. When obtusifolin was administered orally to OA-induced mice daily, the degree of damaged cartilage was dose-dependently reduced. Similarly, the OARSI grade, subchondral bone plate thickness, and osteophyte formation were decreased. These results show that obtusifolin inhibits OA progression.

In summary, our results suggest that obtusifolin inhibits the expression of Mmp3, Mmp13, and Cox2, which is increased under OA conditions. According to our results, obtusifolin inhibits the phosphorylation of p65 in the NF-κB protein complex, which appears to block downstream signaling via the NF-κB signaling pathway. Thus, obtusifolin regulates the expression of OA catabolic factors (Figure 4D). Additionally, these results suggest that obtusifolin, similar to other anthraquinone derivatives, can be used as an anti-OA drug compound.

## 4. Materials and Methods

### 4.1. Primary Culture of Mouse Articular Chondrocytes and Animals

Mouse chondrocytes isolated from femoral condyles and tibial plateaus were obtained from 5-day-old Institute of Cancer Research mice as described previously [5,8]. The cells were cultured in Dulbecco’s modified Eagle’s medium supplemented with 10% fetal bovine serum and 1% penicillin/streptomycin, and grown in an incubator maintained at 37 °C in a 5% CO_2_ atmosphere. C57BL/6 mice (male; 10 weeks old; weighing 18–20 g; *n* = 5) were purchased from DBL (Chungcheongbuk-do, Eumseong, Korea). All the animal experiments were approved by the Animal Care and Use Committee of the University of Ajou. 

### 4.2. In Vitro Chondrocyte Treatment and Reagents

IL-1β, purchased from GenScript (Z02922-10; Picataway, NJ, USA), was dissolved in sterilized water. Obtusifolin and celecoxib were purchased from Sigma-Aldrich (obtusifolin, PHL83880; celecoxib, PZ0008; St. Louis, MO, USA). Primary mouse chondrocytes were incubated at 37 °C with 5% CO_2_ for 4 days. Thereafter, the cells treated with IL-1β (1 ng/mL) were co-treated with obtusifolin for 24 h and harvested on day 5. The cells were also co-treated with celecoxib and IL-1β (1 ng/mL). To identify signaling pathways regulated by obtusifolin, chondrocytes were pretreated with obtusifolin for 24 h. IL-1β (1 ng/mL) was added to the cells, and they were incubated for 10 min in serum-free medium before harvest. 

### 4.3. Cell Cytotoxicity Assay (Lactate Dehydrogenase Assay)

The lactate dehydrogenase (LDH) assay is commonly used to measure cell toxicity [44]. Primary chondrocytes were treated with obtusifolin (0, 25, 50, 100, and 200 μM) for 24 h. The supernatant was then collected and analyzed using an LDH-cytotoxicity assay kit (K311-400; BioVision, Inc., Milpitas CA, USA). We measured the values according to the manufacturer’s recommendation. 

### 4.4. Transcription and Protein Analyses 

Chondrocyte RNA was isolated using TRIzol (Molecular Research Center Inc., Cincinnati, OH, USA) and chloroform. The proteins and lipids in the cells were disrupted with TRIzol, and the RNA was separated by layering with chloroform. The total RNA isolated from the primary chondrocytes was reverse transcribed and used to synthesize cDNA using the ImProm-II™ Reverse Transcriptase kit (A3803; Promega, Madison, WI, USA). The primers and temperature conditions used for each gene are summarized in Table S1. The transcript levels were quantified by qRT-PCR (StepOnePlus Real-Time PCR System; ABI, Beverley, UK). For Western blotting, the proteins were extracted and lysed using lysis buffer (1% NP-40, 50 mM Tris, 5 mM NaF, and 150 mM NaCl) that contained a cocktail of protease inhibitors (25178620; Roche cOmplete™, Madison, WI, USA). The extracted proteins were separated by size using an acrylamide gel, and the primary antibodies used after transfer were as follows: mouse anti-Erk (sc-514302; Santa Cruz, Dallas, TX, USA), rabbit anti-Cox2 (ab52237; Abcam, Cambridge, UK), rabbit anti-P65 (8242S; Cell Signalling Technology), rabbit anti-pP65 (3033S; Cell Signalling Technology), rabbit anti-JNK (9252S; Cell Signalling Technology), rabbit anti-p-JNK (9251S; Cell Signalling Technology), rabbit anti-p38 (cst9212; Cell Signalling Technology), and rabbit anti-pp38 (cst92159; Cell Signalling Technology). Erk was used as the loading control. The band intensities were quantified by densitometry (AlphaEase FC 4.0; Alpha Innotech, San Leandro, CA, USA).

### 4.5. Collagenase Activity and PGE_2_ Assay

The PGE_2_ level was measured using a PGE_2_ immunoassay kit (KGE004B; R&D Systems, Minneapolis, MN, USA). The level of PGE_2_ secreted from primary mouse chondrocytes treated with obtusifolin was measured according to the manufacturer’s protocol. Collagenase activity was quantified using the EnzChek Gelatinase/Collagenase Assay Kit (e12055; Invitrogen, Carlsbad, CA, USA) using the conditioned media of chondrocytes treated with obtusifolin and IL-1β (1 ng/mL). 

### 4.6. Experimental OA-Induced Mice and Oral Administration

Experimental OA was induced by DMM (Destabilization of the medial meniscus) surgery in 10-week-old mice, following a previously reported procedure [5,45]. Sham-operated mice were used as controls. Four weeks after DMM surgery, the mice were orally administered obtusifolin (10, 50, and 100 mg/kg) for 6 weeks. Obtusifolin was dissolved in 90% 1× PBS (CBP007B; LPS), 5% DMSO (D2650-5X5ML; Sigma-Aldrich, St. Louis, MO, USA), and 5% Tween 80 (9005-65-6; Sigma). Only 1× PBS was administered to the control group. 

### 4.7. Histology Analysis

Mouse cartilage samples obtained from DMM-treated mice were fixed in 4% paraformaldehyde and decalcified in EDTA for 2 weeks. They were then embedded in paraffin and cut into 5 μm-thick sections. The sectioned joint samples were stained with Safranin O using a standard protocol [44,46].

### 4.8. Statistics 

The values are presented as mean ± SEM. Statistical significance was estimated using Student’s *t*-test and one-way ANOVA with Dunnett’s post hoc multiple-comparison test. All the analyses were performed using GraphPad Prism 7 (GraphPad, San Diego, CA, USA). The histological data were quantified based on ordinal grading systems, such as the OARSI grade, subchondral bone plate thickness score, and osteophyte grade, using nonparametric statistical methods. The statistical tests were conducted by calculating the probabilities directly from our results. The numbers of repeated experiments and mice used are indicated in the figures.

## Data Availability

The data used to support the finding of this study are included within the article.

## Supplementary Materials

The following is available online at https://www.mdpi.com/1424-8247/14/3/249/s1: Table S1. PCR primers.
