# Supplementary material for: Obtusifolin, an Anthraquinone Extracted from Senna obtusifolia (L.) H.S.Irwin & Barneby, Reduces Inflammation in a Mouse Osteoarthritis Model
Source: Pharmaceuticals (Basel). 2021 Mar 10;14(3):249. doi: 10.3390/ph14030249 (PMC7999271; doi:10.3390/ph14030249)
Supplement: Supplementary file 1 [file pharmaceuticals-14-00249-s001.pdf]

Table 1. Primer sequences and qRT-PCR conditions.

Gene	Origin	Strand	Sequence	Size (bp)	AT[a] (°C)
Mmp3	Mouse	S	5'-CTGTGTGTGGTTGTGTGCTCATCCTAC-3'	350	58
		As	5'-GGCAAATCCGGTGTATAATTCACAATC-3'		
Mmp13	Mouse	S	5'-TGATGGACCTTCTGGTCTTCTGG-3'	473	58
		AS	5'-CATCCACATGGTTGGGAAGTTCT-3'		
Cox2	Mouse	S	5'-GGTCTGGTGCCTGGTCTGATGAT-3'	724	65
		As	5'-GTCCTTTCAAGGAGAATGGTGC-3'		
Gapdh	Mouse	S	5'-TCACTGCCACCCAGAAGAC-3'	450	55
		As	5'-TGTAGGCCATGAGGTCCAC-3'		
qMmp3	Mouse	S	5'-TCCTGATGTTGGTGGCTTCAG -3'	102	60
		AS	5'-TGTCTTGGCAAATCCGGTGTA -3'		
qMmp13	Mouse	S	5'-CTTCTTCTTGTTGAGCTGGACTC -3'	173	60
		AS	5'-CTGTGGAGGTCACTGTAGACT -3'		
qCox2	Mouse	S	5'-TTCAACACACTCTATCACTGGC -3'	271	60
		AS	5'-AGAAGCGTTTGCGGTACTCAT -3'		

[a]AT, annealing temperature; [b]S, sense primer; [c]As, antisense primer
